# Molecular dynamics modeling of the influence forming process parameters on the structure and morphology of a superconducting spin valve

**DOI:** 10.3762/bjnano.11.160

**Published:** 2020-11-24

**Authors:** Alexander Vakhrushev, Aleksey Fedotov, Vladimir Boian, Roman Morari, Anatolie Sidorenko

**Affiliations:** 1 Modeling and Synthesis of Technological Structures Department, Institute of Mechanics, Udmurt Federal Research Center, Ural Division, Russian Academy of Sciences, Baramzinoy 34, Izhevsk 426067, Russia; 2 Nanotechnology and Microsystems Department, Kalashnikov Izhevsk State Technical University, Studencheskaya 7, Izhevsk 426069, Russia; 3 Orel State University named after I.S. Turgenev, Komsomolskaya Str. 95, 302026, Orel, Russia; 4 Ghitu Institute of Electronic Engineering and Nanotechnologies, Academiei Str. 3/3, MD-2028, Chisinau, Republic of Moldova; 5 Laboratory of Topological Quantum Phenomena in Superconducting Systems (TQPSS), MIPT, Institutskiy Per. 9, 141701, Dolgoprudny, Russia; 6 Technical University of Moldova, 168 Stefan cel Mare Avenue, MD-2004, Chisinau, Republic of Moldova

**Keywords:** hybrid nanostructure, mathematical modeling, modified embedded-atom method, molecular dynamics, spintronics, spin valve, vacuum deposition

## Abstract

This work is a study of the formation processes and the effect of related process parameters of multilayer nanosystems and devices for spintronics. The model system is a superconducting spin valve, which is a multilayer structure consisting of ferromagnetic cobalt nanolayers separated by niobium superconductor nanolayers. The aim was to study the influence of the main technological parameters including temperature, concentration and spatial distribution of deposited atoms over the nanosystem surface on the atomic structure and morphology of the nanosystem. The studies were carried out using the molecular dynamics method using the many-particle potential of the modified embedded-atom method. In the calculation process the temperature was controlled using the Nose–Hoover thermostat. The simulation of the atomic nanolayer formation was performed by alternating the directional deposition of different composition layers under high vacuum and stationary temperature conditions. The structure and thickness of the formed nanolayers and the distribution of elements at their interfaces were studied. The alternating layers of the formed nanosystem and their interfaces are shown to have significantly different atomic structures depending on the main parameters of the deposition process.

## Introduction

Multilayer superconductor/ferromagnetic (S/F) hybrid nanostructures are a new type of quantum electronics elements based on electron spin transport. Unlike conventional electronics, spintronics uses not only charge transfer, but also the electron spin in solids, solving the problem of transport and recording of information [1–7]. Based on the basic nondissipative elements of spintronics, it is possible to create new superconducting nanoelectronics devices that consume minimum energy and have a high operation speed [8–13].

One type of magnetic nanostructure with wide potential use is the spin valve [14–15], consisting of several magnetic films separated by a magneto-resistive layer. Due to the exchange interaction with the adjacent antiferromagnetic nanofilm, one of the layers has constant magnetization. For the adjacent nanofilm, the direction of magnetization can be controlled by an external magnetic field. The weak link of the ferromagnetic layers causes a restructuring of the magnetic moment configuration under low-power magnetic fields and switches the spin valve from a high to a low resistance state. When a superconducting film is used as a magneto-resistive interlayer, a superconducting spin valve is obtained. Furthermore, these structures are highly sensitivity to magnetic field switching and energy consumption is significantly reduced due to the absence of dissipation in such a valve in the ground (superconducting) state.

**Figure 1:**
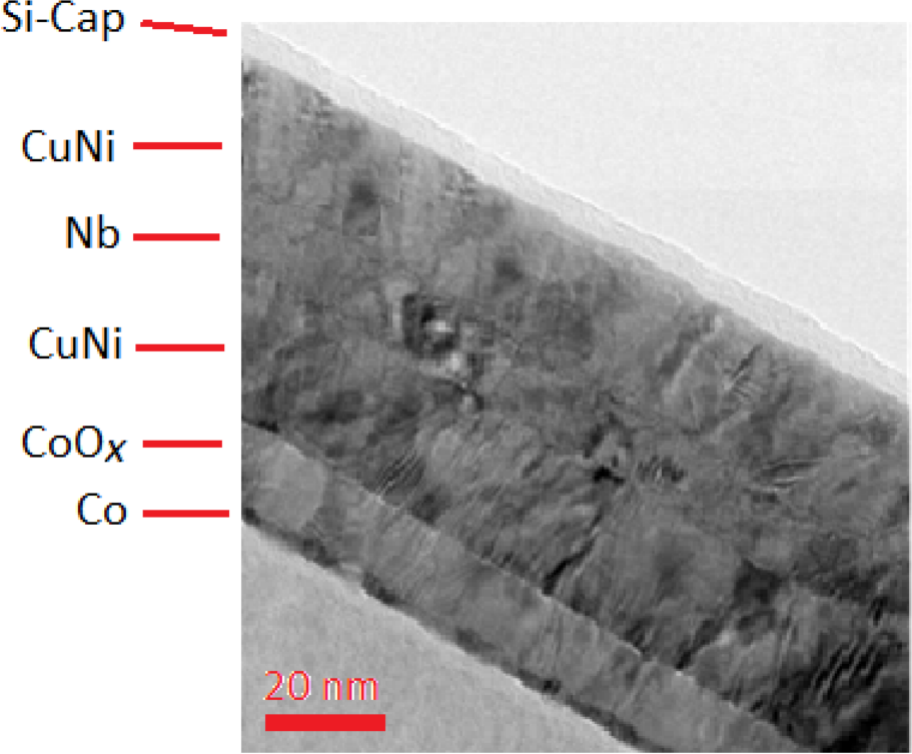
Transmission electron microscopy (TEM) image of a layered nanostructure consisting of Nb, CuNi, CoO*_x_* and Co layers, prepared by magnetron sputtering.

**Figure 2:**
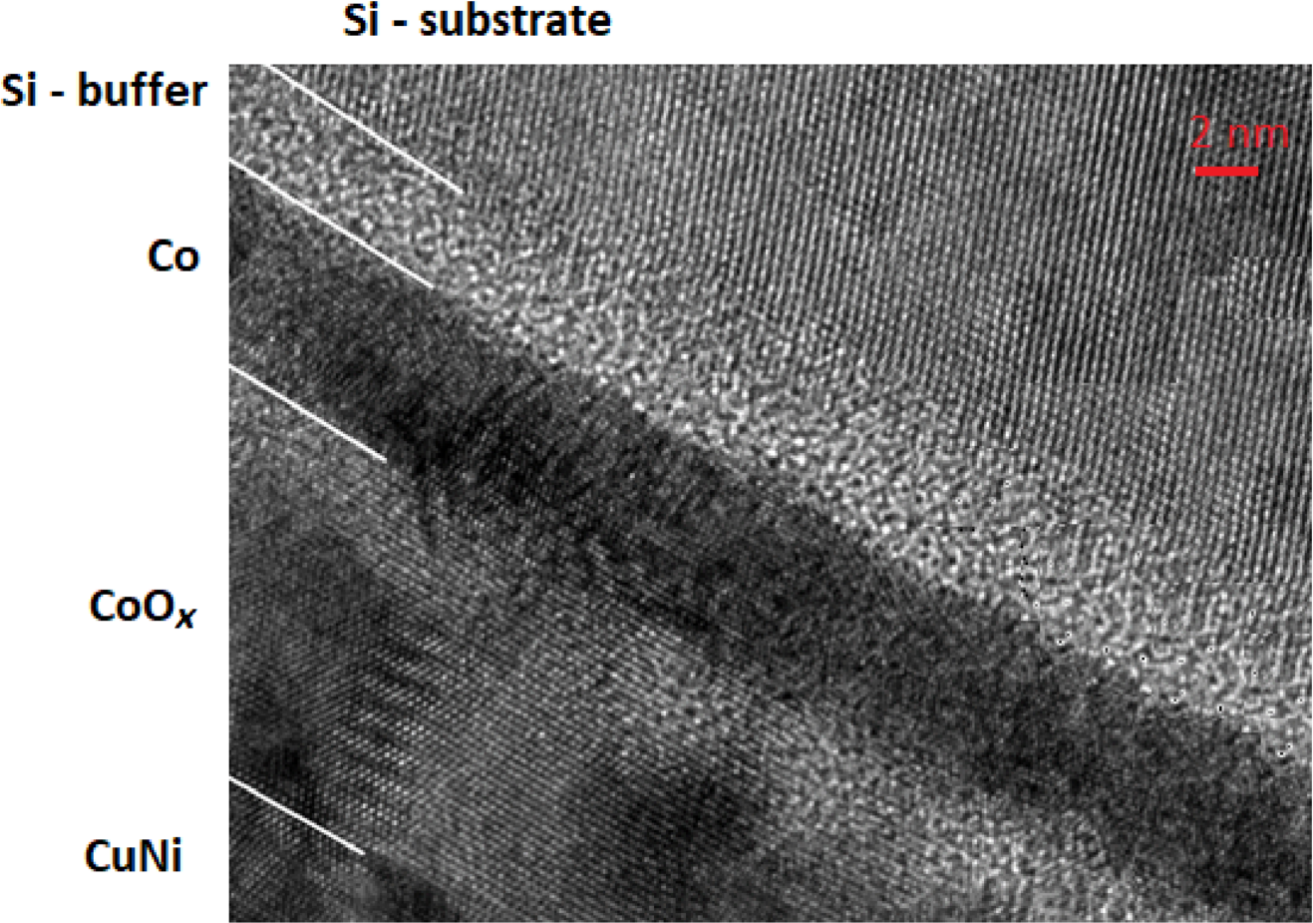
High-resolution transmission electron microscopy (HR-TEM) image of a layered Co, CoO*_x_* and CuNi nanostructure prepared by magnetron sputtering.

Practice shows that the creation of multilayer S/F nanostructures with the required properties is an extraordinarily complex process. Figure 1 and Figure 2 show actual spin-valve multilayer nanosystems formed from various materials [9]. As demonstrated in the figures, the structure of real nanosystems is far from ideal. In particular, it can be noted that the surface separating the various nanolayers of the system is not perfectly flat. The surface has noticeable irregularities that penetrate into adjacent layers. The figures also show that there is a mutual penetration of one contact layer into another. Therefore, the layer interface has a certain quantifiable thickness. It should be noted that the atomic structure of each layer does not form an ideal single crystal, but rather a polycrystalline system is formed.

The influence of the interface quality on the spin valve functionality is an especially important issue, which has been experimentally investigated in previous works [16–19] where the quantum-mechanical transparency of the interface, *T*_F_, was assigned. Here, they considered the effect of the mutual solubility of the metals (of a superconductor and a ferromagnet) on the quantum-mechanical transparency. The transparency parameter of the interface for completely non-wetting metals, such as lead–iron, is small: *T*_F_ ≈ 0.4. This reduces the probability of penetration of Cooper pairs from the superconductor into the ferromagnetic material and requires smaller thicknesses of superconducting layers in order to obtain a significant effect of ferromagnetism on superconductivity. In turn, the structural quality of superconducting niobium layers, for example, with a thickness comparable to the coherence length of approximately <10 nm, is worse than that of thicker films, and the destructive effect of the interface roughness also suppresses the manifestation of interference effects in ultrathin films.

For highly mutually soluble metals, such as vanadium and iron (solubility of about 30% at room temperature), the quantum-mechanical transparency parameter is many times higher, *T*_F_ ≈ 1.6. However, if the structural quality of the films requires deposition on a substrate heated to 100–300 °C, there is a high risk of mutual diffusion and the formation of a thick “dead” layer, which also suppresses the transparency of the S/F interface [20].

In the case of materials wetting and limited mutual solubility (as in niobium–nickel and niobium–cobalt, with solubility of about 5% at room temperature), the transparency parameter is *T*_F_ ≈ 2 and is the highest of all possible metal pairs. It is possible that high transparency is facilitated not only in the mixing layer at the interface between extremely thin film materials, but also by a good matching of the band structures of two metals (see the study in [21–22]). A detailed discussion of the boundary resistance issue of two metals in contact and experimental data analysis for a number of magnetic and nonmagnetic metals pairs is given in [23].

A number of previous studies suggest that a significant increase in the interdiffusion of niobium and cobalt with an increase in the substrate temperature will decrease the transparency parameter *T*_F_ and worsen the functional parameters of the layered S/F heterostructure.

The implementation of optimal technological processes is required to minimize these defects and imperfections of layered nanosystems. This would require basic research for a deep understanding of the physical and chemical processes taking place at different structural levels of the used materials. In addition, the development of manufacturing technology for a fundamentally new device for superconducting spintronics requires a long period of equipment time and highly specialized knowledge, together with a large number of experiments aimed at optimizing the process.

Thus, due to the laborious nature of vacuum technologies, the high cost and long duration of experimental methods for investigation of the physics and chemical properties of S/F nanosystem formation, it would be very useful to develop new integrated methods that combine theoretical modeling and experimental methods for analyzing the formation processes and properties of this class of functional nanomaterials and nanostructures. Here, computer simulation can significantly reduce the number of technological steps and adjustments required to obtain the desired multilayer nanostructure.

It should be noted that mathematical modeling is widely used in the design and analysis of various nanosystems properties [24–25]; however, in relation to the considered class of multilayer nanosystems for spintronics, the number of works is extremely limited.

This work presents the advancements in our previous studies on the modeling of various nanosystems [26–29], including spintronic studies [30–32], where the aim was to investigate the influence of selected technological parameters (substrate temperature, concentration and spatial distribution of the deposited atoms over the interface) on the structure and morphology of the layered nanosystem.

## Mathematical Model and Theoretical Foundations

The formation processes and the structure of multilayer systems for spintronics applications were studied by the molecular dynamics method [33–34]. Molecular dynamics describes the motion of each nanosystem atom at a certain point in time, therefore, it is possible to reproduce the detailed evolution of nanoelements and their properties. The basis of the method is the equations of motion of all atoms supplemented by the initial conditions in the form of atom coordinates and velocities:

[1] $$\begin{array}{l} m_{i}\frac{{\text{d}}^{2}r}{\text{d}t^{2}}=-\frac{\partial U\left(r\right)}{\partial r_{i}}+F_{\text{ex}},\;r_{i}\left(t_{0}\right)=r_{i0},\;\frac{\text{d}r_{i}\left(t_{0}\right)}{\text{d}t}=V_{i0},\\ i=1,...N\;, \end{array}$$

where *N* is the number of atoms that form the nanosystem. In this equation, *m_i_* is the mass of the *i*th atom; **r***_i_*_0_, **r***_i_*(*t*) are the initial and current radius vector of the *i*th atom, respectively; *U*(**r**) is the system potential, which depends on the relative position of all atoms; **V***_i_*_0_, **V***_i_*(*t*) are the initial and current speed of the *i*th atom, respectively; **r**(*t*) = {**r**_1_(*t*), **r**_2_(*t*), …, **r***_N_*(*t*)} shows the dependence of the location of all the atoms in the system; **F**_ex_ is the external force, which reflects the interaction of the nanosystem with the external environment, including adjustments to the energy to maintain a constant temperature.

The molecular dynamics method is based on the concept of potential *U*(**r**), which is responsible for the nature and magnitude of the interactions of the atoms in the nanosystem.

There are many possible choices for the type of potential, but recently, due to its accuracy and adequacy, many-particle force fields have gained great popularity. In this work, we used the potential of the modified embedded-atom method (MEAM) which is based on density functional theory (DFT). In this method, the resulting potential of the nanosystem is represented as the sum of the energy contributions of the individual atoms, and the contributions of pair and multielement interactions are considered separately. Thus,

[2]
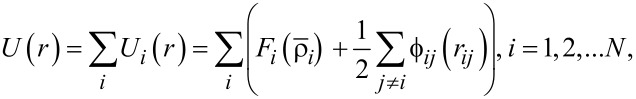


where *U_i_*(*r*) is the potential of an individual atom, affecting the type and degree of interaction in the equations of motion (Equation 1); *F_i_* is the atom immersion function, which is dependent on electron background density 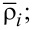 ϕ*_ij_*(*r_ij_*) is a contribution of the pair potential to the total energy, which varies with distance *r_ij_*.

The immersion function is corrected by the force field created through pair interactions and refines this value. This value is due to the presence of electron gas in the material and, in accordance with [33–34], can be calculated by the formula

[3] $$F_{i}\left({\bar{\rho }}_{i}\right)=\{\begin{array}{c} A_{i}E_{i}^{0}{\bar{\rho }}_{i}\ln\left({\bar{\rho }}_{i}\right),\;{\bar{\rho }}_{i}\geq 0\\ -A_{i}E_{i}^{0}{\bar{\rho }}_{i},\;{\bar{\rho }}_{i}<0\;\;\;\;\;\;\;\;\;\;\; \end{array},$$

where *A_i_* is an empirical force field parameter; 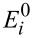 is the value of sublimation energy; and 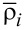 is the value of the background electron density.

To calculate the background electron density at the immersion point, the following equation is used in which all electronic orbitals of atoms of various configurations contribute their terms as

[4] $${\bar{\rho }}_{i}=\frac{\rho_{i}^{\left(0\right)}}{\rho_{i}^{0}}G\left(\Gamma_{i}\right),\;\;\Gamma_{i}=\sum_{k=1}^{3}t_{i}^{\left(k\right)}{\left(\frac{\rho_{i}^{\left(k\right)}}{\rho_{i}^{\left(0\right)}}\right)}^{2},$$

where the parameters 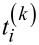 are the weight coefficients of the model; 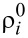 is the magnitude of the background electron density of the initial structure; and 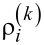 characterizes the change in electron density under real conditions. The indices *k* = 1,2,3 represent the different types of electronic orbitals of an atom. In this sense, there are spherically symmetric one-electron s-orbitals and angular-electron p-, d-, and f-clouds. The electronic distribution density of each orbital is calculated according to its own formula:

[5] $$s\text{ orbital: }\rho_{i}^{\left(0\right)}=\sum_{i\neq j}\rho_{j}^{A\left(0\right)}\left(r_{ij}\right)S_{ij},$$

[6]
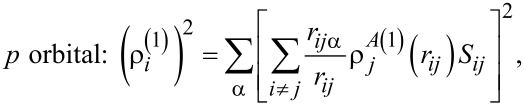


[7]
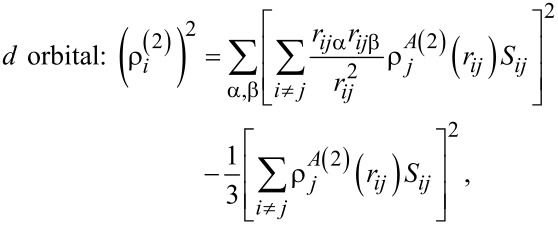


[8]
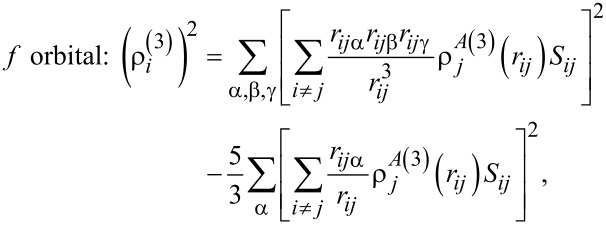


where ρ*^A^*^(^*^k^*^)^ are radial functions; *S_ij_* is the potential shielding function; and *r_ij_*_α_ is component α from the distance vector between atoms α,β,γ = *x*,*y*,*z*.

The functional *G*(Γ) in Equation 4 can be defined in various ways. One of the most popular formulations is used here and is given as follows:

[9] $$G\left(\Gamma \right)=\{\begin{array}{c} \sqrt{1+\Gamma },\;\Gamma \geq -1\\ -\sqrt{\left|1+\Gamma \right|},\;\Gamma <-1 \end{array}.$$

The weight coefficients of the MEAM from Equation 4 also have an additive relationship with single-electron radial functions given as

[10]
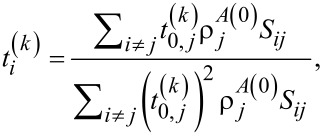


where 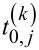 are empirical parameters that depend on the chemical type of the element.

Distance energy smoothing in MEAM is achieved by introducing a shielding function. Using the screening function, the attenuation of the potential occurs gradually, which allows one to provide a more physically accurate nanomaterial properties description and reduces computational costs during simulation:

[11] $$\begin{array}{l} S_{ij}=f_{\text{c}}\left(\frac{r_{\text{c}}-r_{ij}}{\Delta r}\right)\prod_{k\neq i,j}f_{\text{c}}\left(\frac{C_{ikj}-C_{\min,ikj}}{C_{\max,ikj}-C_{\min,ikj}}\right),\\ C_{ikj}=1+2\frac{r_{ij}^{2}r_{ik}^{2}+r_{ij}^{2}r_{jk}^{2}-r_{ij}^{4}}{r_{ij}^{4}-{\left(r_{ik}^{2}-r_{jk}^{2}\right)}^{2}}, \end{array}$$

[12] $$f_{\text{c}}\left(x\right)=\{\begin{array}{c} 1,\;x\geq 1\;\;\;\;\;\;\;\;\;\;\;\;\;\;\;\;\;\;\;\;\;\;\;\;\;\\ {\left[1-{\left(1-x\right)}^{4}\right]}^{2},\;0<x<1\\ 0,x\leq 0\;\;\;\;\;\;\;\;\;\;\;\;\;\;\;\;\;\;\;\;\;\;\;\;\; \end{array},$$

where *C*_min_, *C*_max_ are the parameters of the mutual influence of atoms, depending on their chemical types, and are set for each triple of atoms with numbers *i*, *j*, *k*. In Equation 11, *r*_c_ is the distance at which the force field is cut off and *f*_c_(*x*) is a function that smooths the potential after *x* exceeds the cutoff distance *r*_c_.

## Problem Statement and Software

The influence of the process parameters on the formation of spin system S/F hybrid structures is studied for a multilayer nanosystem based on cobalt and niobium. This system is a functional material that has demonstrated a giant spin-valve effect, which was theoretically and experimentally investigated in previous works [13,19,35]. In these works, the authors proposed a new design and performed the calculation of a spin valve consisting of superconducting plates and an artificial magnetic metamaterial placed between them, formed by periodically alternating thin and thick nanolayers of a ferromagnetic metal. The thickness of the layers affects the magnetic exchange interaction between ferromagnetic layers, which allows for the possibility of artificial magnetic metamaterials designed with tunable properties.

The choice of niobium and cobalt as the metals forming the nanolayers is made because of the wide potential of using these elements in spintronics. At the moment, not only has research been carried out on spintronic devices involving these metals [36–37], but also new patents are being issued [38–40].

**Figure 3:**
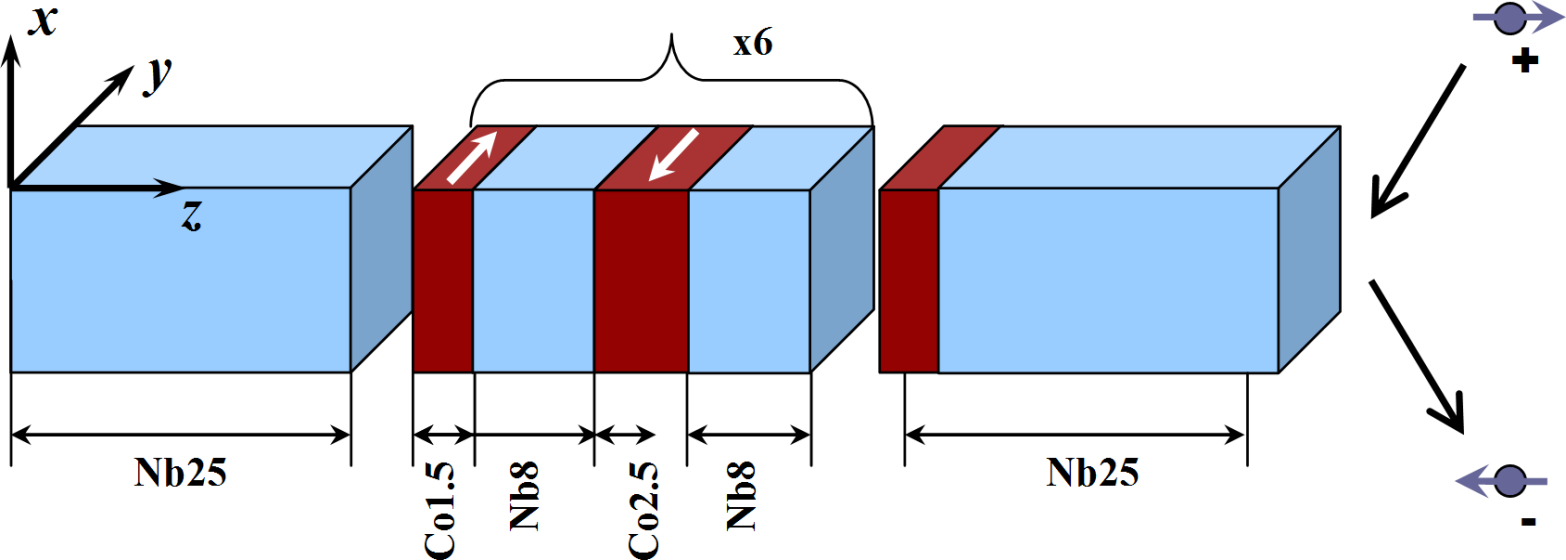
Sketch of a Nb/Co spin-valve nanosystem. The numbers next to the elements in the layers represent the layer thickness in nanometers.

The general scheme of the investigated nanosystem is presented in Figure 3.

The numbers in Figure 3 next to the elements in the layers represent their thickness in nanometers. The sample production is carried out by the magnetron deposition method in vacuum. In general, a nanosystem contains about 20 layers. In this work, we consider the deposition of only the first four layers of cobalt and niobium.

**Figure 4:**
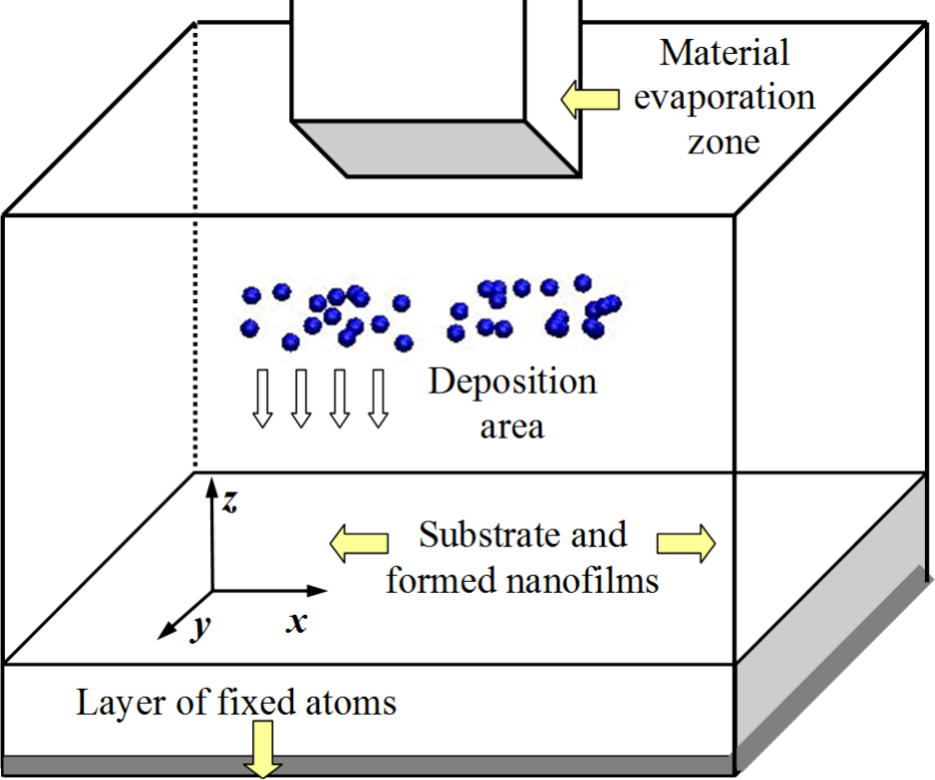
Scheme of the multilayer nanosystem formation modeling processes. The nanosystem contains a material evaporation zone, atom deposition area, and a substrate with a fixed lower layer of atoms. During the first nanofilm formation, the deposition is carried out directly on the substrate; subsequent depositions are made on the substrate with previously formed nanofilms.

The general problem statement for the multilayer nanosystem formation process modeling is presented in Figure 4. The first material layer formed by atoms of single-crystal niobium (in the real deposited structures niobium layers have crystalline structure [18]) serves as the substrate and the basis for the vacuum deposition of subsequent nanofilms.

The substrate is placed in the lower region of the computational cell; its extreme layer is fixed to prevent chaotic movement of the sample during the simulation. In the horizontal direction, periodic boundary conditions are imposed on the computational cell, which reduces the computational cost. In the upper region, boundary reflection conditions are present so that the deposited atoms do not leave the modeling system. The deposition process is simulated by the appearance of atoms in the evaporation zone above the substrate. In this case, the deposited atoms gained speed towards the substrate. The layers are sprayed in stages. During the formation of all layers, the magnetic field was absent in the nanosystem.

The temperature and pressure for the deposited atoms was not regulated. The deposited atoms are given an initial velocity of approximately 0.1 Å/ps (or 10 m/s) towards the substrate surface. Subsequently, the speed of these atoms gradually decreases due to the energy exchange with the substrate surface atoms and the upper atom’s layers of the formed nanolayers. The substrate temperature in each calculation is held constant. The concentration of the deposited atoms was about five atoms per cubic nanometer.

The upper boundary of the computational cell was shifted during the transition to the deposition of the next nanolayer by the value of its thickness. Thus, the deposition region above the substrate turned out to be approximately the same for each nanosystem layer. The process parameters that affect the resulting nanomaterial properties were the following: the deposition rate, controlled through the number of deposited atoms per unit time; substrate temperature; and spraying flux density, which was determined by the area of the evaporation zone.

As the computational module of the program for theoretical research, the large-scale atomic/molecular massively parallel simulator (LAMMPS) computing complex was used [41]. This software and tool package is freely distributed, contains the ability to perform parallel computing and supports multilevel mathematical models, including molecular dynamics. The results analysis algorithms were described in TCL and C++. Based on LAMMPS, scripts and algorithms were developed and implemented for a detailed study of the structure and spatial profile of a S/F material. The results were visualized using the Visual Molecular Dynamics (VMD) [42] and Open Visualization Tool (OVITO) [43] software packages, which not only provide images of nano-objects atomic and molecular structures, but also construct spatial profiles and distributions by the target parameter, e.g. height or coordination number.

## Results and Discussion

A series of numerical experiments on modeling the multilayer hybrid structure formation processes based on cobalt and niobium were performed. The variable elements in the studies were the material fabrication technological parameters, including the substrate temperature, the intensity and area of the deposition flow. The influence of technological modes was evaluated in comparison with the basic version of the nanosystem.

As a basic variant, the growth processes were considered at 300 K (substrate temperature) and deposition was carried out by a uniform flow over the entire surface of the substrate. The term temperature in this work is synonymous with substrate temperature.

The temperature of the nanosystem was controlled using a Nose–Hoover thermostat. The deposited atoms had a directed velocity; therefore, they were not involved in the direct correction of the thermostat. The formation of a multilayer system was carried out in several stages. Each layer was deposited by the sequential deposition of niobium or cobalt.

At the first stage, cobalt was deposited on a substrate formed by niobium atoms. The substrate had a crystalline structure with a height of 3.7 nm and a width of 13.2 nm in the horizontal direction. For the mark of zero height, from which the layers of the deposited material began to form, the substrate surface was chosen. The number of niobium atoms in the substrate was 33,600.

**Figure 5:**
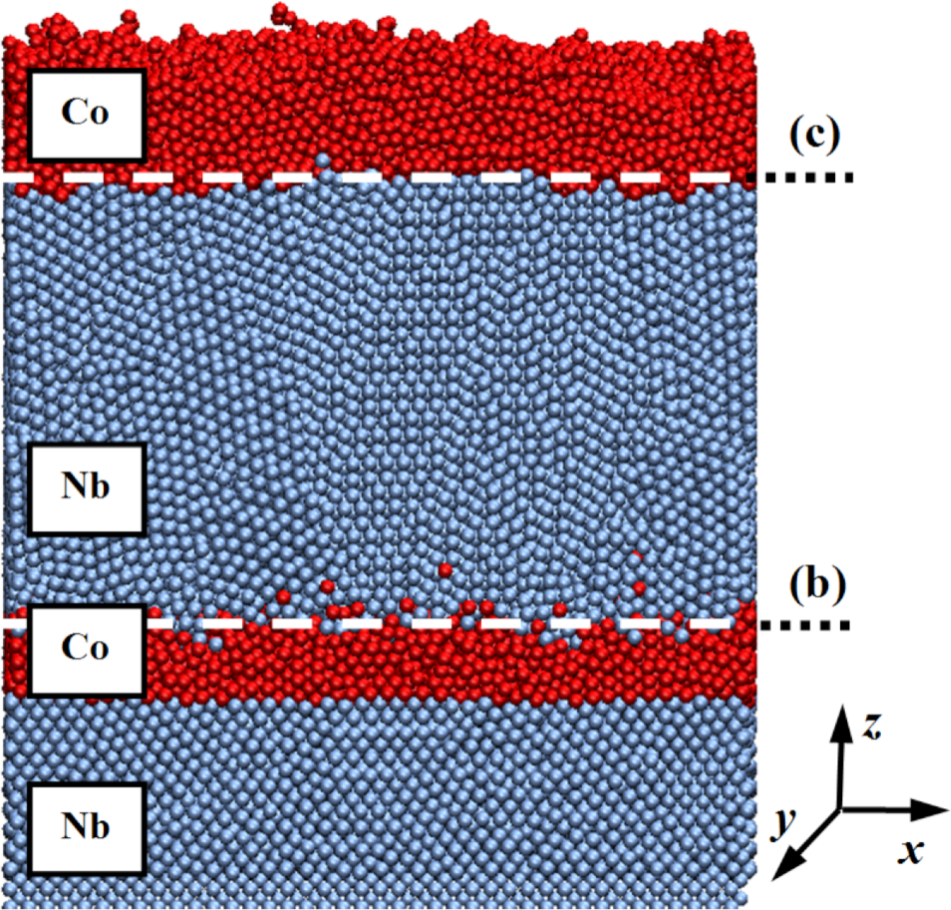
Multilayer nanosystem of niobium and cobalt. The contact points of the nanofilms are indicated by (b) and (c). At the indicated contact points, the distribution of coordination numbers is given in Figure 6. The substrate temperature was fixed at 300 K.

To match the simulation results and experimental data, in addition to the required nanolayer thickness, 18,000 cobalt atoms were deposited on the substrate in the first stage, 70,000 niobium atoms were deposited in the second, and 30,000 cobalt atoms were deposited in the third stage. As a result, three nanofilms with a thickness of 1.5 nm, 8.0 nm and 2.5 nm were formed. The duration of the layer deposition process under normal conditions was chosen according to the desired thickness and was 0.2 ns, 0.6 ns, and 0.4 ns, respectively. An image of a multilayer nanosystem resulting from the mathematical modeling is presented in Figure 5.

Figure 5 clearly demonstrates the niobium and cobalt layered nanosystem formation: the layers formed by niobium and cobalt atoms have a polycrystalline structure. In this case, groups of atoms are combined into domains with different spatial orientations. The blurring of the contact area between the layers and a less even surface profile compared to niobium are noticeable.

**Figure 6:**
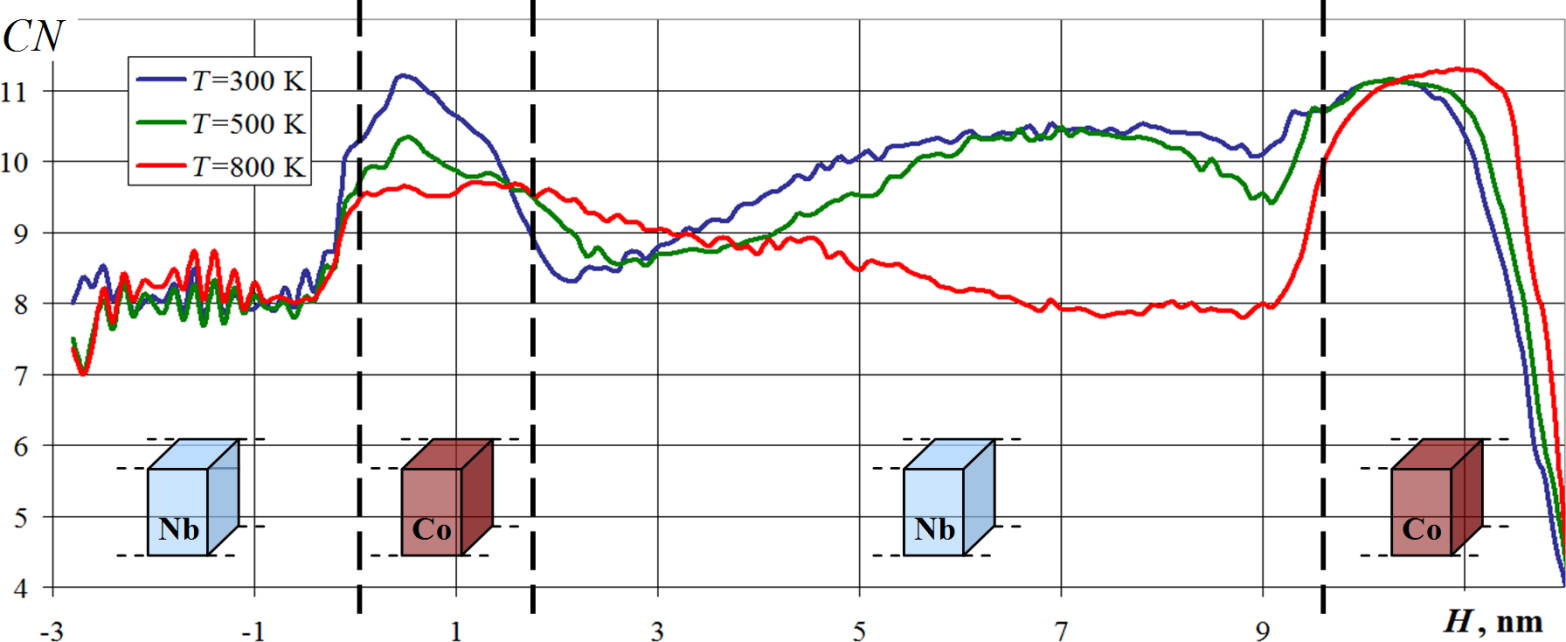
Change in coordination (CN) number along the *z*-axis (shown in Figure 5) in Nb and Co layers of the nanostructure. The contact points of the nanofilms are shown by the dashed vertical lines. The distribution is plotted for substrate deposition at 300, 500, and 800 K.

A quantitative characteristic of the material spatial structure can be obtained by calculating the coordination number. The coordination number in crystallography reflects the number of nearby equally distant atoms of the same type in the crystal lattice. The number of nearest neighbors determines the material packing density. For different types of crystal lattices, the coordination number will be different. The cubic volume-centered lattice (characteristic of niobium) has a coordination number equal to 8, whereas the hexagonal close-packed lattice (corresponding to cobalt) is 12. For the formed nanosystem, the change in the average value of the coordination number in the layers was calculated and shown in Figure 6.

The change in the coordination number in Figure 6 correlates with the structure of the nanomaterial shown in Figure 5. The niobium substrate has a parameter value close to 8, which indicates its crystalline structure. The cobalt nanofilms are characterized by a higher coordination number in the range of 10–11. This value does not reach 12, which corresponds to the ideal crystalline state of a hexagonal close-packed lattice, indicating an amorphous-like structure of cobalt nanofilms. Variations in the coordination number within the intermediate niobium layer are more significant. When approaching the contact regions with cobalt, an increase in this parameter is observed.

**Figure 7:**
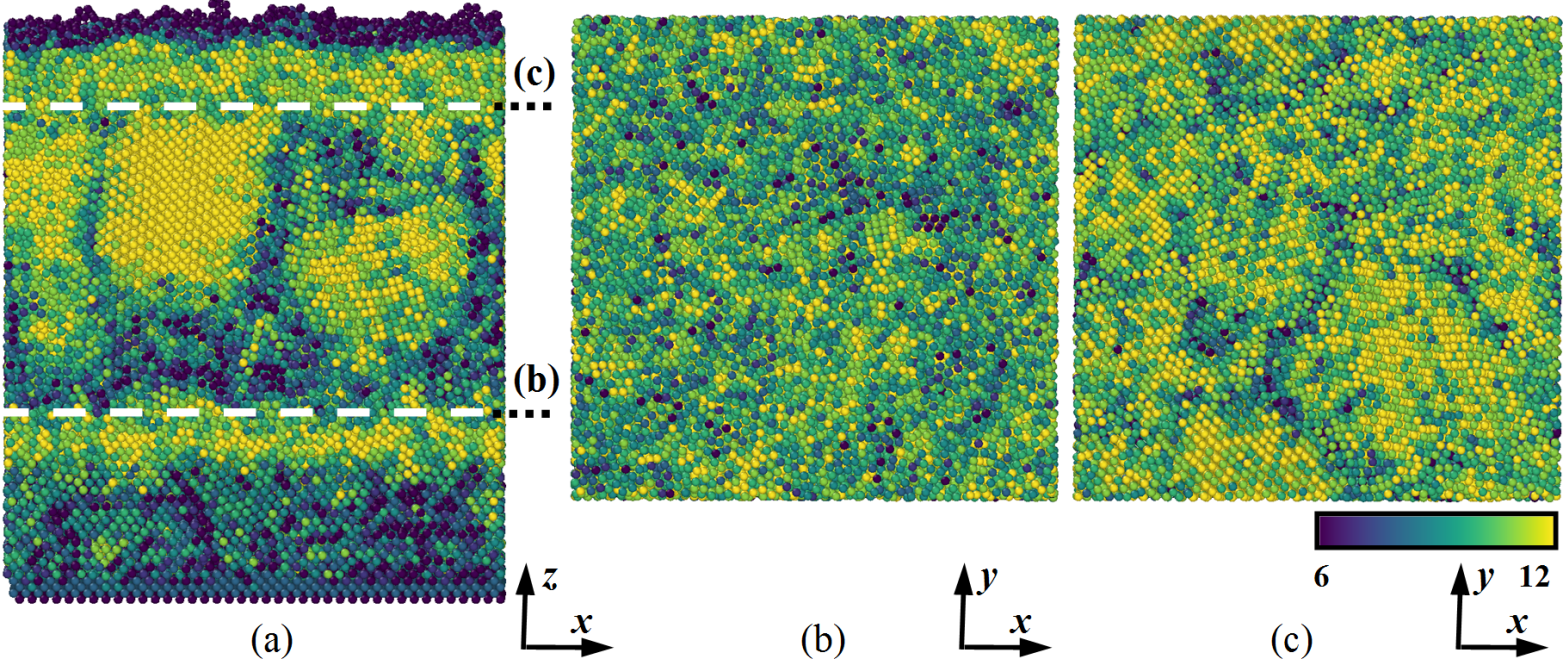
Spatial distribution of the coordination number in the formed multilayer Nb–Co (a) system and in its parallel sections (b) and (c). Substrate temperature is 300 K.

Thus, it was shown that the structure of the nanomaterial depends not only on the current layer characteristics, but also on the structural features of the regions adjacent to it. In addition, the temperature has a definite effect on the number of nearest neighbors in a nanosystem, and therefore on its structure and properties. A significant decrease in the coordination number in the outer layers of the last nanofilm is associated with the surface effects and boundary phenomena appearance in that region. The spatial distribution of this parameter is shown in Figure 7.

The spatial distribution of the coordination number in the formed multilayer nanocomposite, illustrated in Figure 7, characterizes its structure in more detail. The dashed lines in Figure 7 indicate the locations of the parallel sections shown in Figure 7b and Figure 7c. The sections correspond to the contact zones of the nanolayers and are also marked in Figure 5. The color profile of the coordination number corresponds to an increased value for cobalt layers. The value of the parameter in these layers is variable with a spread over a certain range of values. The niobium substrate has a lower coordination number. The structure of this region was initially crystalline and changed insignificantly during modeling and deposition. An interesting effect is observed in the intermediate niobium nanolayer, where distinct crystallization zones appeared. Crystallization zones have a higher coordination number and are characterized by a denser packing of atoms. The described regions arise in sufficiently thick films mainly near cobalt layers. The initial metal crystal lattice mismatch causes mutual rearrangements of atoms and structure transformation inside the material.

**Figure 8:**
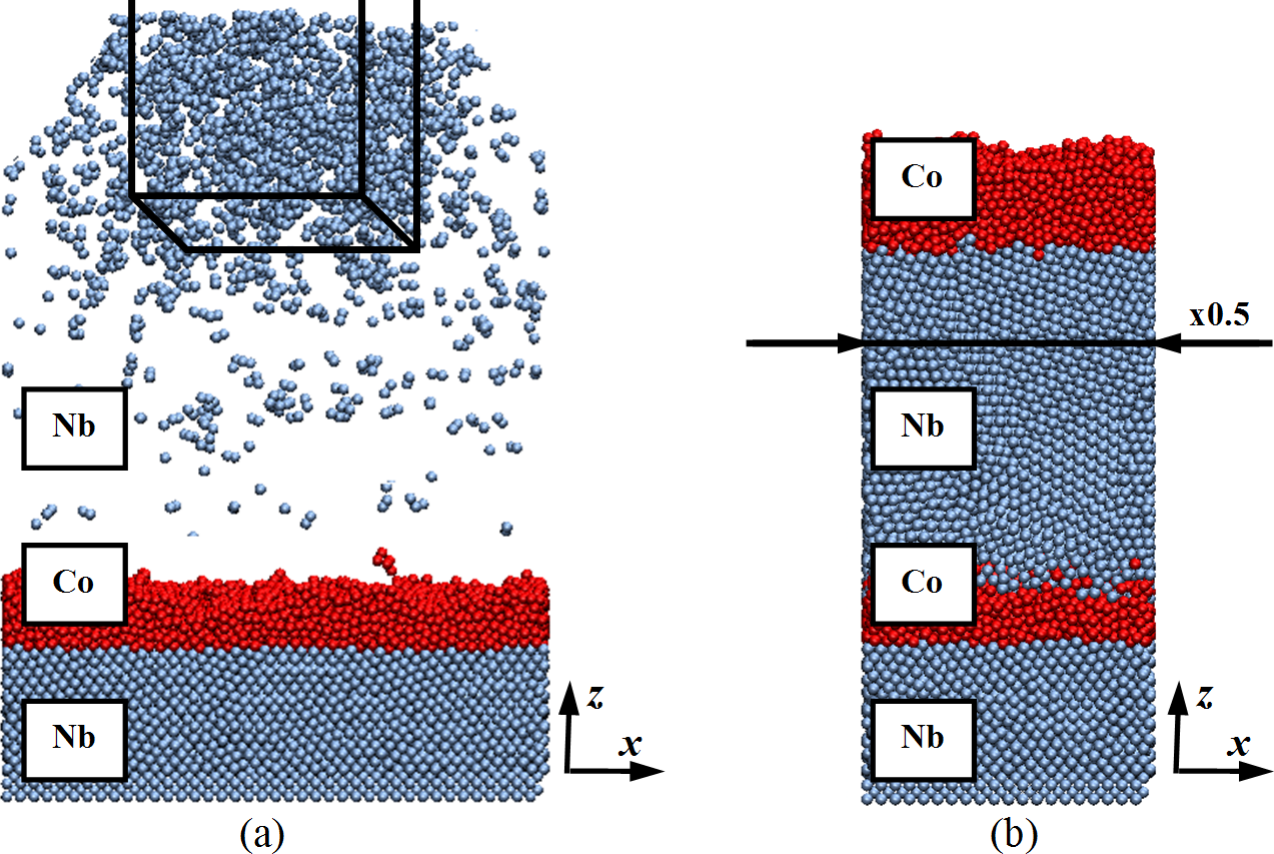
The area of deposition flow (a) and the size of the simulation area (b). The deposition flow area is shown at the top of (a) with solid black lines. The narrowing of the computational domain in (b) is illustrated by the factor ×0.5 in the horizontal direction.

The next series of computational experiments was aimed at understanding the deposition flux area influence and the modeling region size on the structure and morphology of the simulated layered nanosystem. Figure 8 shows these parameters of the nanosystem.

The change in the area of the deposition flow, illustrated in Figure 8a, was carried out by reducing the evaporation zone of the starting materials shown in the upper region of this figure. The spray flow area was reduced four times from the original value.

Also, nanocomposite formation modeling was performed on a scale reduced by four times. In this case, the number of deposited atoms in each layer was proportionally reduced so that the thickness of the formed nanofilms did not change.

**Figure 9:**
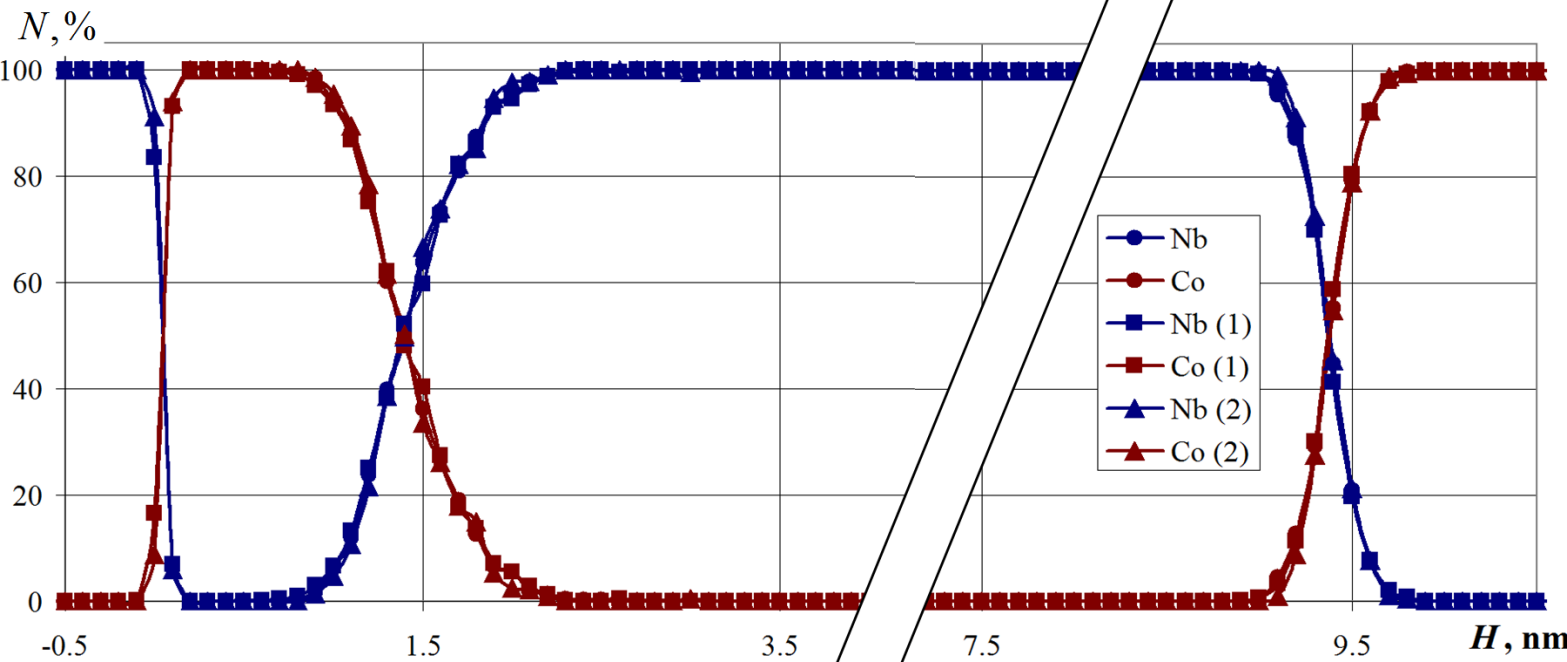
Relative layer-by-layer composition of the Nb–Co nanosystem when the deposition flux is reduced by four times (1) and the modeling area is reduced by four times (2). The parameter *N* is the cobalt or niobium percentage in the horizontal nanofilm layers. The substrate temperature is 300 K.

The deposition flow area influence and the modeling region size on the relative layer-by-layer nanosystem composition are shown in Figure 9. The dependencies without an index in parentheses correspond to the nanosystem formation in its basic mode. The analysis of this result reveals a decrease in the evaporated metal content and a decrease in the calculated region, which does not lead to the rearrangement of the active atoms or a change of the nanolayer composition. A decrease in the area of the deposition flux led only to the increase in the atomic density zone in the upper region above the substrate. Leaving the evaporation zone, the deposited atoms tend to occupy a more energetically favorable state and are scattered in an evenly distributed layer throughout the entire free volume. When the deposited atoms reach the substrate surface, the effect of reducing the flux is leveled.

A decrease in the nanosystem transverse size by a factor of four also did not affect the nanosystem layer composition, which can be seen in Figure 9. The dependence of the niobium and cobalt fractions slightly differ from the basic version of the nanosystem.

Changing the size of the simulation area allows us to analyze how well the computational cell is represented. Small computational domains can lead to incorrect simulation results due to the appearance of boundary effects. As follows from the calculations, a four-fold decrease in the volume of the nanosystem did not affect the nanocomposite layer composition. The layers are connected at the same places, and the proportion of niobium and cobalt differ slightly from the main part of the sample. The obtained data indicate that the initially selected modeling area is fully representative, and the results properly reproduce the properties of the modeled nanosystem.

The next series of computational experiments was aimed at elucidating the dependence of the multilayer nanosystem structure on the flux density of deposited atoms. This value is controlled by an increase or decrease in the number of deposited atoms per unit time introduced into the system from the evaporation zone. Here, numerical calculations for the nanofilm variations with a 2-fold increase and a 1.5-fold decrease of the deposited atom flux density are performed.

**Figure 10:**
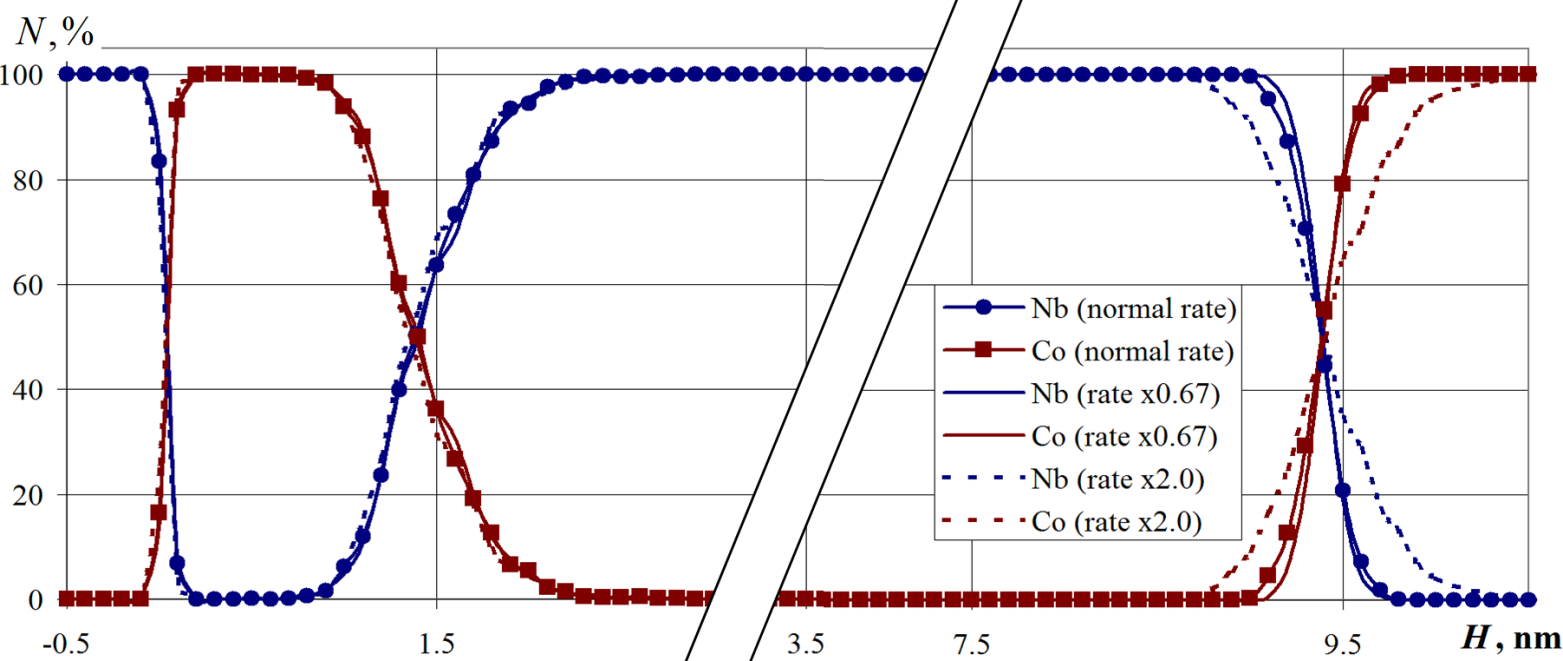
The relative layered composition of the Nb–Co nanosystem for different deposition rates. The parameter *N* is the cobalt or niobium percentage in the horizontal nanofilm layers. The substrate temperature is 300 K.

The relative layered composition of the nanocomposite for the calculations is shown in Figure 10. Here, the fraction of elements in the composition during the nanolayer formation when the deposition rate was reduced by 1.5-fold is shown by the solid lines without markers, the increased intensity is represented by dashed lines without markers and the base atom deposition intensity is represented by solid lines with markers. The analysis of Figure 10 reveals that a decrease in the deposition rate of the metals did not significantly affect the composition distribution within the layers of the multilayer nanosystem.

An increase in speed led to a deviation of the composition from the data obtained in the basic version of the calculation. A significant increase in the flux intensity leads to higher agglomeration of the metal atoms above the surface of the substrate. The structure of the resulting nanofilms directly depends on the size of the deposited clusters and does not always have time to rebuild upon direct contact with the surface. Due to the effects that arise, inhomogeneity, dislocations, and voids can occur in the material. The deviations in the constructed composition in the upper layers of the nanocomposite were due to a more rarefied structure, and additional mixing of the nanofilm contact regions is especially noticed. The conducted studies indicate that there is a critical deposition rate, whereby a higher rate leads to the formation of a nanomaterial with a different structure. Since in real technological processes the deposition is carried out using a sufficiently low intensity (about 1000 nm per hour), in order to obtain physically adequate results, the deposition process must be simulated at a speed not exceeding this critical value. On the other hand, there is no need to increase the duration of the nanofilm growth stage. The maximum should approximate the true experimental value, and as given in Figure 10, the structure and composition in this case are similar.

**Figure 11:**
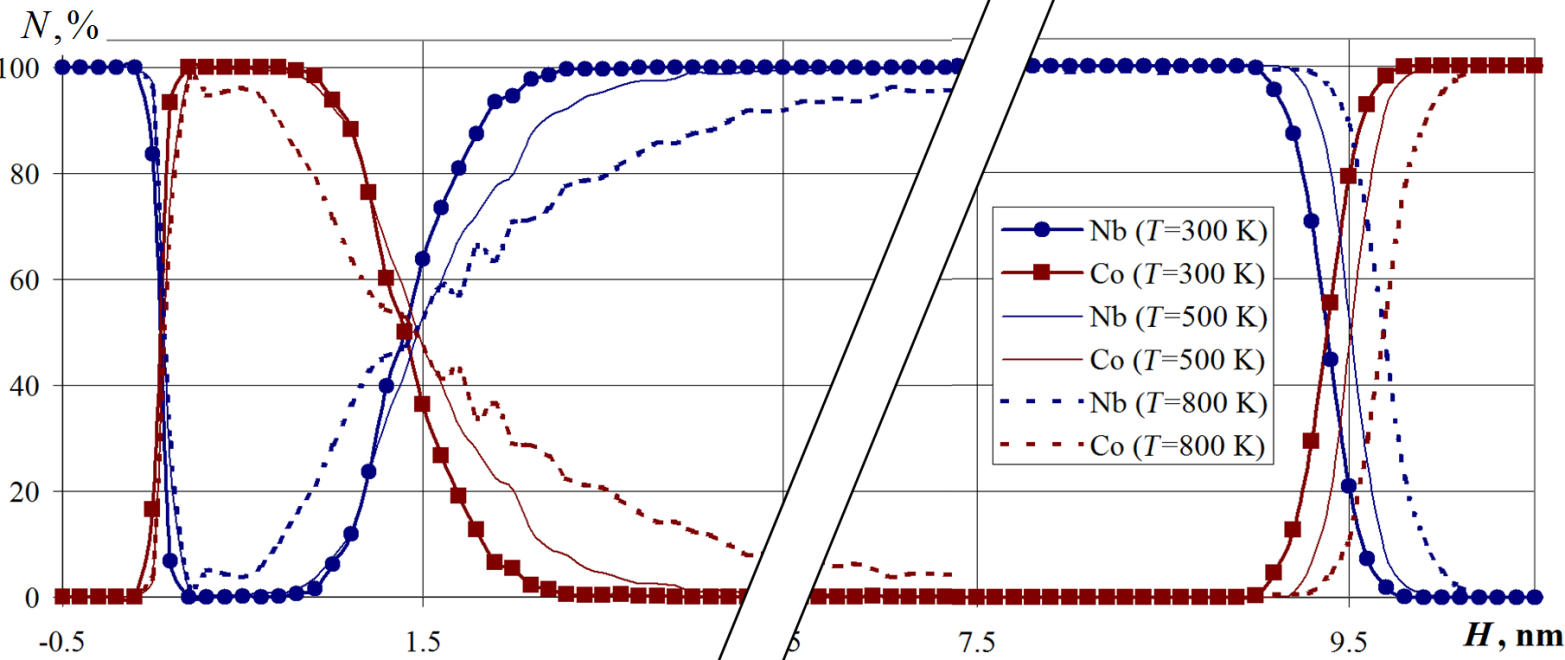
The percentage composition of the Nb–Co multilayer nanosystem formed at a substrate temperature of 300, 500, and 800 K. The parameter *N* is the cobalt and niobium percentage in the horizontal nanofilm layers.

A series of computational experiments was carried out in which the multilayer Nb–Co nanosystem formation was studied in the temperature range of 300–800 K for substrate temperatures of 300, 500, and 800 K, respectively. The simulation results are presented in Figure 11 in the form of a percentage composition of the nanosystem.

The calculations showed that the temperature of the substrate significantly affects the nanosystem structure formation. An increase in temperature leads to an increase in the total thickness of the nanosystem (at 800 K, this value is increased by 0.3 nm compared with a temperature of 300 K). The region of mutual penetration of Nb atoms into the Co layers (and vice versa) also increases, which is clearly seen in Figure 11.

Noticeable variations in the nanosystem layer composition at the interface regions of all nanolayers are visible. The obtained results indicate the multilayer nanosystem formation processes vary significantly including the atomic structure of the interface contact areas, as well as the multilayer nanosystem composition and structure due to the increase in the thermal diffusion processes.

## Conclusion

The paper proposes a technique and describes a mathematical model for studying technological modes and parameters in the manufacture of multilayer nanosystems. The model was tested in the study of the formation of a nanosystem based on a hybrid niobium and cobalt structure for a superconducting spin valve design. The influence of various technological parameters was investigated including substrate temperature, deposition flow rate and density, and the nanosystem dimensions.

An analysis of the coordination number distribution in the material showed that the layers have a different structure when multilayer nanofilms are formed under normal conditions. The niobium substrate structure is close to crystalline; cobalt nanofilms are characterized by an amorphous-like structure. In the thickened niobium layer, crystallization zones are observed where direct contact with the cobalt nanolayer occurs. The crystal lattice mismatch of the starting metals causes mutual rearrangements of atoms and the structure transformation inside the nanosystem.

It was also found that a decrease in the area of the deposition flux and the simulation region by 75% of the initial value does not lead to atomic rearrangement or to a change in the nanofilm composition. A decrease in the deposition flux area caused an increase in the atomic density zone in the upper region above the substrate.

The adjustment in the number of vaporized atoms per unit time and the duration of the nanofilm deposition stages revealed the layer and contact area compositional dependence on the intensity of the starting element deposition. A significant increase in the deposition flux intensity led to the appearance of inhomogeneities, dislocations and voids inside the formed nanosystem due to the preliminary clustering of free atoms.

In order to obtain representative results that correspond to real technological processes, it was found to be critical to model the growth mechanisms of nanoscale films and layers using a deposition rate not exceeding the critical value of clustering.

It was also found that the substrate temperature is a leading factor affecting the multilayer nanosystem formation, the atomic structure of the interface contact areas, as well as the composition and structure of the multilayer nanosystem as a whole.

The results presented in this work can be used as a tool to support future experimental investigations and for adjusting and optimizing technological processes related to the production of multilayer nanosystems and devices for spintronics.
